# Laser spectroscopic probing of coexisting superfluid and insulating states of an atomic Bose–Hubbard system

**DOI:** 10.1038/ncomms11341

**Published:** 2016-04-20

**Authors:** Shinya Kato, Kensuke Inaba, Seiji Sugawa, Kosuke Shibata, Ryuta Yamamoto, Makoto Yamashita, Yoshiro Takahashi

**Affiliations:** 1 Department of Physics, Graduate School of Science, Kyoto University, Kyoto 606-8502, Japan; 2 NTT Basic Research Laboratories, NTT Corporation, Atsugi 243-0198, Japan

## Abstract

A system of ultracold atoms in an optical lattice has been regarded as an ideal quantum simulator for a Hubbard model with extremely high controllability of the system parameters. While making use of the controllability, a comprehensive measurement across the weakly to strongly interacting regimes in the Hubbard model to discuss the quantum many-body state is still limited. Here we observe a great change in the excitation energy spectra across the two regimes in an atomic Bose–Hubbard system by using a spectroscopic technique, which can resolve the site occupancy in the lattice. By quantitatively comparing the observed spectra and numerical simulations based on sum rule relations and a binary fluid treatment under a finite temperature Gutzwiller approximation, we show that the spectra reflect the coexistence of a delocalized superfluid state and a localized insulating state across the two regimes.

## Abstract (web summary)

A system of ultracold atoms in an optical lattice can be used as a quantum simulator for the Hubbard model with high controllability. Here, the authors report a laser spectroscopy study of an ytterbium ultracold bosonic gas across the weakly to strongly interacting regime.

## Graphical abstract

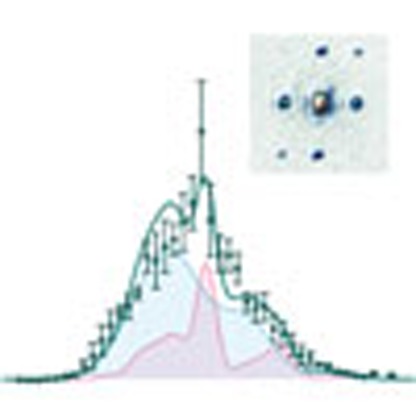

Ultracold atoms confined in an optical lattice potential offer a novel way to study quantum many-body physics^1^,^2^. One of the most interesting problems is the quantum phase transition of ultracold bosonic atoms in a three-dimensional (3D) optical lattice from a superfluid (SF) state to a Mott insulating state. Various experimental techniques such as matter-wave interference^3^,^4^,^5^, noise-correlation measurements^6^,^7^ and quantum gas microscopes^8^,^9^,^10^ are used to probe important statistical quantities such as phase coherence, the density-density correlation and the atom number distribution, which characterize the quantum phase transition. On the other hand, various spectroscopic measurement techniques such as Bragg spectroscopy^11^,^12^,^13^, lattice modulation spectroscopy^14^,^15^,^16^ and radio-frequency (RF) spectroscopy^17^,^18^ offer unprecedented potential for studying the dynamical response of interacting atoms as a result of an artificially induced perturbation. Excitation energy spectra measured with high precision allow us to obtain a deeper insight into many-body effects beyond the static and statistical observables. Furthermore, and impressively, high-resolution laser spectroscopy has recently revealed the novel SU(N) symmetry^19^,^20^,^21^.

In this paper, we report a laser spectroscopic study of the ultracold bosonic atoms in a 3D optical lattice across the weakly to strongly interacting regimes. By probing the very different on-site interactions between the different electronic states with the optical transition (^1^S_0_→^3^P_2_) of an ytterbium (Yb) atom, our method is excellent for resolving different site occupancies, in both strongly^18^,^19^,^20^ and weakly interacting regimes. To understand the excitation spectrum quantitatively, we develop a numerical method for calculating the spectrum, which is based on a finite temperature Gutzwiller approximation and formally derived sum rule relations^22^. The great and continuous change in the observed spectra as regards their shapes across the two regimes indicates that the spectroscopy captures the complicated change in the many-body state with high sensitivity, and a comparison of the spectra and the numerical simulations provides a comprehensive understanding of the observation.

## Results

### Theoretical framework for the laser spectroscopy

Before excitation, the electronic ground ^1^S_0_ state atoms in an optical lattice are in thermal equilibrium, which is described by the many-body ground-state Hubbard Hamiltonian:


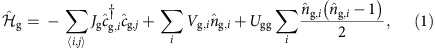


where 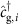 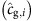 is the creation (annihilation) operator of an Yb atom in the electronic ground ^1^S_0_ state at the *i*-th site and 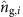 is its number operator, and the summation 〈*i*,*j*〉 is taken over the nearest-neighbour sites. *J*_g_, *V*_g,*i*_ and *U*_gg_ represent the hopping energy, the trapping potential at the *i*-th site determined from the Gaussian beam shape of the trapping lasers and the on-site interaction energy, respectively.

The single orbital model well describes the low energy properties of atoms before excitation except for very shallow lattices, whereas the higher orbitals are inevitably involved after excitation. The excitation operator 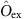 is written as 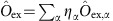 with second quantization. Here 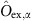 describes the orbital-changing excitations from the lowest to the *α*th bands, and is given by


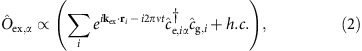


where *ν* and **k**_ex_ are the excitation laser frequency and wave vector, and 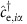 is the creation operator of the electronic excited ^3^P_2_ state atoms in the *α* orbital at the *i*-th site of a position **r**_*i*_. *η*_*α*_ is defined by 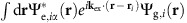, where 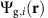 and 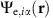 are the Wannier orbitals of the states corresponding to 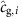 and 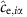, respectively. An excitation probability from the lowest to the *α* th orbitals is given by |*η*_*α*_|^2^, where ∑_*α*_|*η*_*α*_|^2^=1.

**Figure 1:**
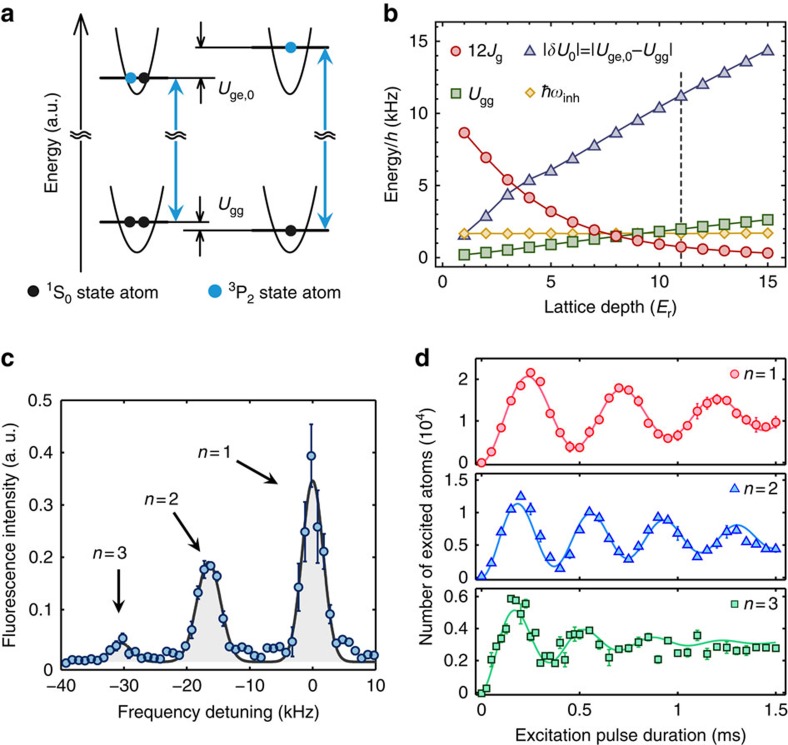
Site-occupancy resolving laser spectroscopy. (**a**) Energy diagram of singly and doubly occupied sites in an optical lattice. The doubly occupied sites have energy shifts due to on-site interactions, which depend on the atomic state. (**b**) The relevant energy scales in the laser spectroscopy (see the text). The vertical dashed line denotes the critical lattice depth of the Mott transition^30^ according to the mean-field theory for a homogeneous system. (**c**) The site-occupancy resolved spectrum in a deep lattice. The large attractive interaction of *U*_ge,0_ gives sidebands corresponding to the excitations in the multiple occupied sites on the negative frequency side. Error bars indicate s.e.m., and the solid line denotes a triple Gaussian fit to the data. Note that we only observe a single resonance peak even for multiply occupied sites due to a collisional blockade effect. (**d**) Separately induced Rabi oscillations. The observed Rabi frequencies are 2.1, 2.7 and 2.8 kHz for the singly, doubly and triply occupied sites, respectively. The lattice depth is 22*E*_r_ in **c**,**d**. The experimental details can be found in Supplementary Note 2.

After the excitation, the Hamiltonian is written as 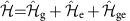, where


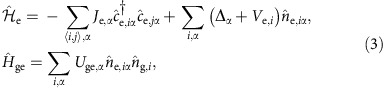


where Δ_*α*_ is the energy difference between the lowest and *α* orbitals, and the other parameters are labelled in the same manner as equation (1). Here *U*_ge,*α*_ represents the on-site interaction of atoms in the electronic ground state and lowest orbital with atoms in the excited state and orbital *α* (Fig. 1a). *V*_e,*i*_ denotes the trapping potential for the electronic excited state atoms, which is generally different from that for the ground-state atoms. Furthermore, the interaction between two electronic excited atoms *U*_ee_ can be neglected under a weak excitation condition.

The excitation energy spectrum *I*(*ν*) is obtained by measuring the number of electronic excited atoms as a function of *ν*. The number conservation law of electronic excited atoms allows us to decompose *I*(*ν*) into the sum of *I*_*α*_(*ν*):


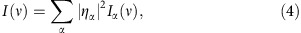


where *I*_*α*_(*ν*) is formally given in a weak excitation limit by Fermi's golden rule as


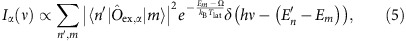


where |*m*〉 represents the many-body eigenstates of the Hamiltonian 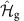 in equation (1) with energy *E*_*m*_, and 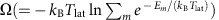 is the grand canonical potential. Here, *k*_B_ is the Boltzmann constant, *T*_lat_ is the temperature after lattice loading and *h* is Planck's constant. The many-body excited state |*n*′〉 with energy 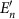 is associated with the Hamiltonian 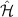. For simplicity, assuming the probe time 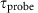 to be infinite, the delta function is used to describe spectrum. Note that spectral broadening caused by the finite probe time and also certain physical origins mentioned below will be taken into account in the numerical simulations.

As it is difficult to calculate the spectrum *I*_*α*_(*ν*) by directly evaluating equation (5), we use the formally derived sum rule relations of spectral moments 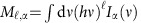 (ref. 23). Here, 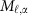 can be given by certain thermodynamic quantities, that is, 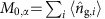, and the explicit forms of *M*_1,*α*_ and *M*_2,*α*_ are found in the Methods. As pointed out in refs 22, 24, the spectral peak position *p*_*α*_ can be determined from the first and zeroth order moments: *p*_*α*_=*M*_1,*α*_/*M*_0,*α*_. For example, at *T*_lat_=0, the number-definite states (NDS) with *m*-atom filling show *p*_NDS,*iαm*_=(*m*−1)*δU*_*α*_+*δV*_*i*,*α*_, where *δU*_*α*_=*U*_ge,*α*_−*U*_gg_ and *δV*_*i*,*α*_=*V*_e,*i*_+Δ_*α*_−*V*_g,*i*_. This feature clearly explains that the present laser spectroscopy can be used to resolve site occupancy^24^.

The second moment allows us to determine the spectral variance 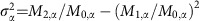. For simplicity, we consider the two limited cases at *T*_lat_=0 in the uniform system, which allows us to obtain a simple form of *σ*_*α*_ from equations (10) and (11) in the Methods. These simple calculations clarify the physical origins of spectral broadening caused by the many-body effects. First, for the local NDS, the long-range correlation can be negligible: 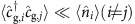. Therefore, we obtain:


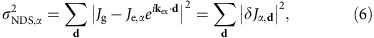


where **d** represents a vector to the adjacent site. The spectrum for NDS is broadened by the tunnelling effects including a momentum transfer of **k**_ex_. In contrast, for the coherent phase-definite states (PDS), where we can use a mean field approximation 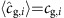, we get the 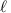-body correlation function 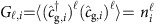 and 
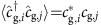
. Then, we obtain:


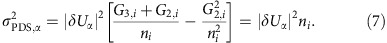


For PDS, the spectrum should be broadened by number fluctuations resulting from the delocalized nature of the coherent state. Equations (6) and (7) show that the definite quantity does not cause spectral broadening, and its conjugate should be an origin of the broadening because of large fluctuations resulting from uncertainty^22^.

In general, at finite temperatures in inhomogeneous systems, phase-definite and number-definite states can coexist, and so we use binary fluid approximation in ref. 22, in which *I*_*α*_(*ν*) is decomposed into two contributions from incoherent normal fluid (NF) and coherent SF atoms *I*_*α*_(*ν*)=*I*_NF,*α*_(*ν*)+*I*_SF,*α*_(*ν*). In addition, by considering the spectral broadening mentioned above, we use the following expressions for the spectral functions: For the NF,


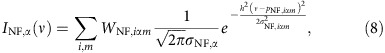


where *p*_NF,*iαm*_(∼*p*_NDS,*iαm*_) and *σ*_NF,*iαm*_(∼*σ*_NDS,*α*_) are the spectral position and width of the *m*-atom-occupying state at *i*th site. The spectral weight is given by 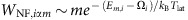, where *E*_*m*,*i*_=*m*(*m*−1)*U*_gg_/2+*mV*_g,i_ and 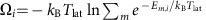. On the other hand, for the SF,


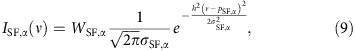


where spectral properties *W*_SF,*α*_, *p*_SF,*α*_ and *σ*_SF,*α*_ are determined from the sum rule relations mentioned in the Methods. Note that because of the finite temperature effects and the correlations between SF and NF, the spectral properties (for example, *p*_NF,*iαm*_ and *σ*_SF,*α*_) are not always equivalent to the simplified forms (for example, *p*_NDS,*iαm*_ and *σ*_PDS,*α*_, respectively). By further considering the spectral broadening caused by the finite probe time 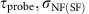 will be replaced as 
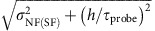
 with 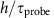 of 1 kHz, which effectively describes the competition between the probe time and the tunnelling time. Supplementary Note 9 and ref. 22 provide more details.

In the numerical calculation, we adopt the finite temperature Gutzwiller approximation to obtain the thermodynamic quantities in the spectral moments 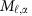. This approximation is a mean-field approximation considering up to the first-order correction in terms of *J*_g_, and then 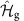 is approximated by a set of the effective local Hamiltonians (Supplementary Note 7). Therefore, on the basis of the local approximation, the onsite 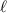-body correlation 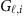 can be obtained by diagonalizing the effective local Hamiltonian, and the long-range correlation 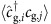 is described by 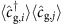.

### Site-occupancy resolving laser spectroscopy

In the experiments, a small portion of the ^1^S_0_ state atoms was directly excited to the ^3^P_2_ state by a narrow-linewidth laser. Since the high detection efficiency of our fluorescence measurement method enables us to detect as few as 10 atoms, we can perform the spectroscopy in a sufficiently weak excitation regime. The spectroscopy method and the pulse sequence are described in detail in the Methods, Supplementary Fig. 1 and Supplementary Note 1. The novel feature of our spectroscopy technique is its extremely high site-occupancy resolving power (Fig. 1). A Yb atom in the ^3^P_2_ state has a significantly different scattering property from a ^1^S_0_ state atom^25^,^26^. Therefore, we can tune the difference of the on-site interactions |*δU*_0_|=|*U*_ge,0_−*U*_gg_| sufficiently large compared with the other energy scales in the Hubbard Hamiltonian^27^, such as *U*_gg_, the hopping term *J*_g_ and the site-dependent inhomogeneous trapping potential *V*_g(e),*i*_ (Fig. 1b). As a result, the optical resonances associated with different site occupancies are well resolved as shown in Fig. 1c. Note that the peaks on the negative frequency side correspond to the doubly and triply occupied sites, and this is confirmed by controlling the total atom number or the site occupancies with the photo-association technique^28^. The coexistence of such peaks demonstrates the inhomogeneous nature of our system with a harmonic trap. In addition, the high site-occupancy resolving power is highlighted by the ability to separately induce Rabi oscillations for different fillings, which also demonstrates the novel controllability of our method, as shown in Fig. 1d.

We should note that as shown in Fig. 1b, the difference |*δU*_0_| exceeds other energy scales included in the Hubbard Hamiltonian over a wide range from the weakly to the strongly interacting regimes. This is in good contrast to the RF spectroscopy of alkaline atoms^18^,^24^,^29^, in which high site-occupancy resolution is available only in a deep Mott insulating regime because of the relatively small difference between the on-site interaction energies of the hyperfine states.

### Spectra in weakly to strongly interacting regimes

**Figure 2:**
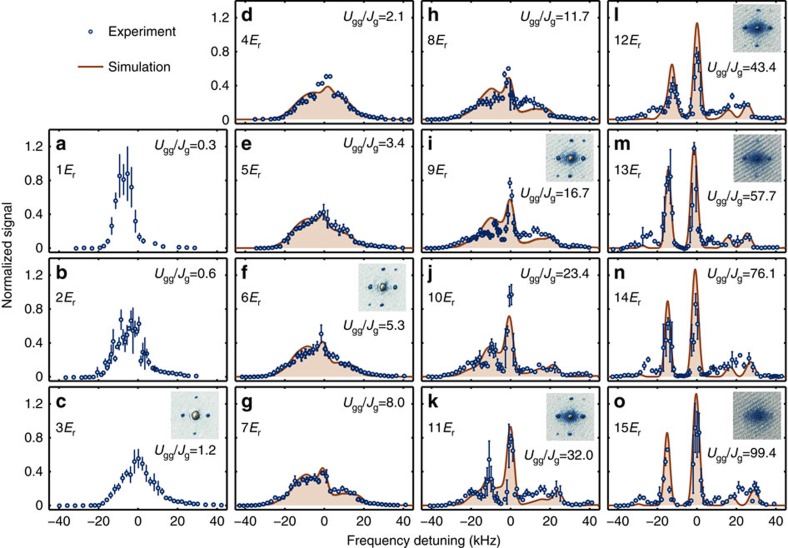
Laser spectroscopy of an atomic Bose–Hubbard system. The lattice depth is changed in 1–15*E*_r_ in which the system covers the weakly to strongly interacting regimes (**a**–**o**). The solid lines denote numerically calculated spectra (see the Methods and Supplementary Note 9). The ratios *U*_gg_/*J*_g_ for each lattice depth are also shown, and the critical (*U*_gg_/*J*_g_)_c_=29.34 (ref. 36) for average filling *n*=1 is determined by the quantum Monte-Carlo method. The light shifts due to the different lattice depths are subtracted by using a linear fit of the resonance frequency of the largest central peaks in the lattice depths at 9 to 15*E*_r_. The zero frequency corresponds to the resonance position of the singly occupied sites, and the spectra are normalized as the area of each spectrum to be unity 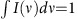. Typically, less than 5 × 10^2^ atoms among about 2 × 10^4^ total atoms are excited to the ^3^P_2_ state, ensuring the weak excitation regime. Circles show experimental observations, and error bars indicate s.e.m. The inset images show absorption imaging results. The imaging is performed after the preparation of atoms in the lattice potential followed by 14 ms ballistic expansion instead of spectroscopy. The field of view is 350 by 350 μm. The excitation laser for the spectroscopy and the imaging axis are perpendicular to the vertical axis, and bisect the angle between the two optical lattice axes on the horizontal plane.

In Fig. 2, we show the spectra *I*(*ν*) observed by widely varying the lattice depth *V*_L_ from 1 to 15*E*_r_, where *E*_r_ denotes the recoil energy associated with the lattice photon. The spectrum without the lattice potential (*V*_L_=0) can be found in Supplementary Fig. 2 and Supplementary Note 3. In Fig. 2, we also show the results of a numerical simulation in which the actual experimental parameters such as the atom number, temperature and inhomogeneous trapping potential are taken into account and there is no fitting parameter except for the normalization factor. The agreement between the numerical calculation and the observed spectra is satisfactory in Fig. 2d–o. In addition, we show the matter-wave interference data in the standard absorption images in the insets in Fig. 2. The shapes of the spectra change greatly compared with the interference peaks, which simply become blurred as the lattice depth is increased.

At lattice depths greater than 11*E*_r_ (Fig. 2k–o), we observe discrete peaks in the spectra, similar to those in Fig. 1c because of the negative interaction energy difference *δU*_0_, and the small peaks on the positive side are resonances including orbital excitation. The discrete nature suggests that the number states are realized in each site, and this can be understood from the fact that the Hubbard parameter *U*_gg_/*J*_g_ exceeds 30 at these lattice depths, and the system enters the strongly interacting regime^30^. Moreover, as shown in Fig. 2d–k, the spectra undergo a complicated change from a single broad peak to several discrete peaks. At a glance, the broad spectra observed in this regime seem to be inconsistent with our site occupancy resolving power as shown in Fig. 1b at relatively deep lattice depths. However, the broad spectrum is a manifestation of the superfluidity in the system, and its width can be explained by the number fluctuation of the SF components in each lattice site (see equation (7)). Furthermore, the coexistence of the discrete and broad peaks directly reflects the coexistence of the delocalization and the localization in the system caused by the effects of the inter-atomic interaction, finite temperature and inhomogeneity^29^,^31^,^32^. It should be noted that the spectral structure also depends on the time scales of the probe and tunnelling (Supplementary Note 10). In this study, the probe time 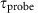
(=1 ms) is longer than or comparable with the tunnelling time of *h*/(12*J*_g_) up to the deep lattice *V*_L_=11*E*_r_. Therefore, the spectrum reflects the detail of the many-body state, such as the SF, NF or their coexistence. The competition between the finite probe time and the tunnelling time is effectively considered in the numerical simulation via the spectral width as discussed above, which allows us to properly calculate the spectra in the strongly correlated regime with a small amount of or no SF component, even though 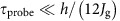. To understand the observed spectra including the coexistence regime quantitatively, we discuss the numerical simulation results in further detail.

### Decomposition of SF and NF contributions

**Figure 3:**
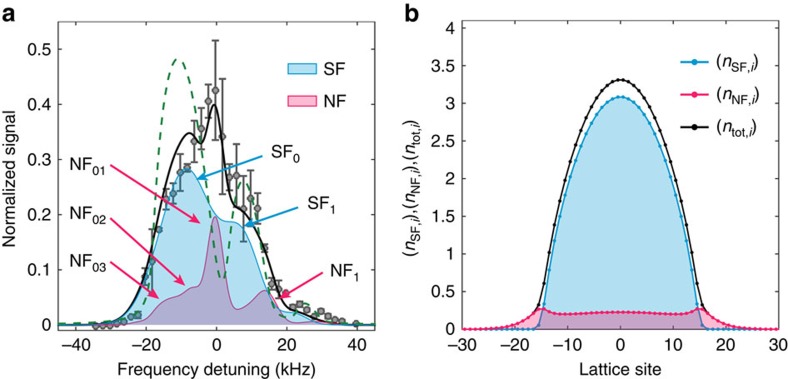
Numerically decomposed contributions in the excitation spectrum and number distribution. (**a**) Contributions to the spectrum from the SF and NF components and (**b**) atom distributions in the optical lattice (*V*_L_=5*E*_r_). The blue and red solid lines correspond to the SF and NF components, respectively, and the black lines are their sum. The green dashed line indicates the calculation result, which includes up to the first-order sum rule relation (see the text). The arrows in **a** labelled SF_*α*_, NF_*αm*_ and NF_*α*_ indicate the corresponding resonance peaks, where *α* and *m* are described in equations (8) and (9), respectively. The grey circles denote the same data shown in Fig. 2 for comparison.

We examine the spectral features in detail by comparing experimental and calculated results. We show the numerically calculated contributions from the SF and NF components separately in Fig. 3. The full results can be found in Supplementary Figs 6 and 7 and Supplementary Note 11. As shown in Fig. 3a (*V*_L_=5*E*_r_) and also Fig. 2d–f (4–6*E*_r_), we find characteristic triangular spectra centred at *ν*=0, and the shape is reasonably explained by the numerically obtained spectra as follows. In such a shallow lattice, the SF atoms are dominant, but the NF atoms with low filling can also be seen in Fig. 3b. The filling *n*_SF,*i*_ is distributed up to three with an average of *n*_ave_ so that the SF contribution *I*_SF_(*ν*) has one broad peak around 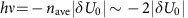 (Fig. 3a, SF_0_) and another broad peak resulting from the orbital excitations around *hν*=−*n*_ave_|*δU*_0_|+Δ_1_ (Fig. 3a, SF_1_). In addition, the NF atoms with low filling contribute to the additional spectral component around *hν*=0 (Fig. 3a, red curve). Thus, the triangular spectra can be understood as a result of the SF-NF coexistence.

The spectral widths for the NF and SF components are determined by equations (6) and (7), respectively, and we also consider the inhomogeneous broadening because of the trapping potential and the excitation laser linewidth. The inhomogeneous broadenings *ħω*_inh_ are almost constant and ∼1.7 kHz in this work (Fig. 1b) and the laser linewidth is ∼1 kHz (Supplementary Fig. 4 and Supplementary Note 5). On the other hand, *σ*_NF,*α*_ and *σ*_SF,*α*_ depend on the Hubbard parameters, hence, the lattice depth. 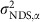 is 13.5|*J*_*g*_|^2^ in our experimental setting and it contributes ∼1.0 kHz to the spectral width for the NF component in Fig. 3a. The spectral width for the SF component depends on *n*_ave_ and is ∼8.0 kHz for the *α*=0 resonance (SF_0_) in Fig. 3a.

For comparison, we also performed a simple calculation at zero temperature with the following expression^24^: 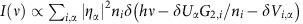, where we substitute *δ*(*ν*) for a Lorentzian with a 1-kHz linewidth. Figure 3a shows an apparent discrepancy between the calculated spectrum (green dashed line) and the experimental results. In particular, a peak at around *ν*=0 is missing, which is the NF contribution within the binary fluid calculations. Note that the large intensity of 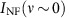 results from the dilute and widely spread distributions of the NF atoms (see Fig. 3b for details).

**Figure 4:**
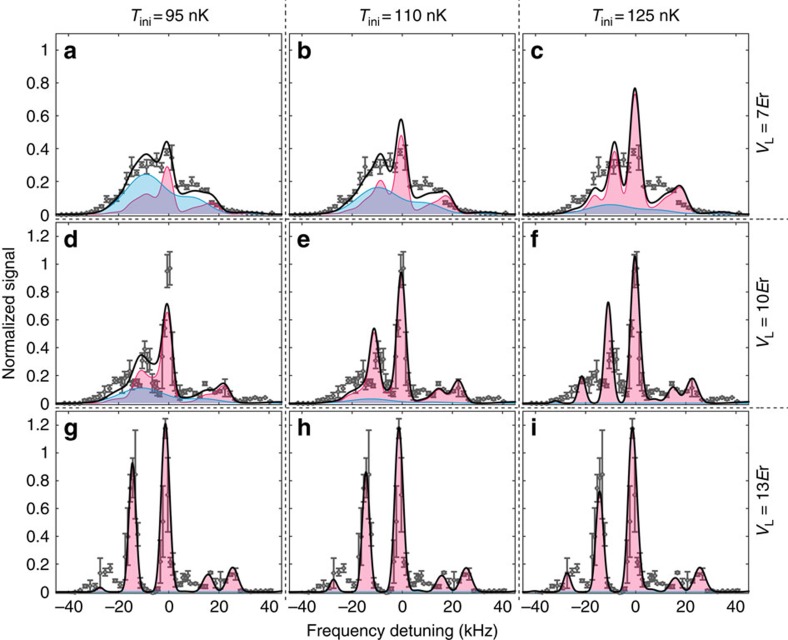
Temperature dependence of the excitation spectra. Contributions to the spectrum from the SF and NF components of different lattice depths and initial temperatures for the calculation. The lattice depths are (**a**–**c**) 7*E*_r_, (**d**–**f**) 10*E*_r_ and (**g**–**i**) 13*E*_r_. The initial temperatures *T*_ini_ for the calculation are (**a**,**d**,**g**) 95 nK, (**b**,**e**,**h**) 110 nK and (**c**,**f**,**i**) 125 nK. The colour scheme is the same as in Fig. 3. The grey circles denote the same data shown in Fig. 2 for comparison.

As the lattice depth is increased further, the triangular shape is distorted and discrete peaks appear that reflect a noticeable increase in the incoherent component *I*_NF_ as shown in both Fig. 4a (*V*_L_=7*E*_r_) and Fig. 2g–k (7–11*E*_r_). In addition, the discrepancy between the experimental and numerical results is more pronounced: the discrete peaks near *ν*=0 are more contrasted in the experimental result (Fig. 2h–k). In the numerical calculation presented in Figs 2 and 3, we assume the adiabatic atom loading into the lattice potential with the initial entropy of *S*_ini_ calculated from the initial temperature *T*_ini_ by neglecting the interaction effects for the Bose gas. Here, *T*_ini_ is determined by a standard time-of-flight measurement and the laser spectroscopy without lattice potential, both of which allow us to calibrate the thermal fraction of Bose gases. To cover non-adiabatic heating effects and also an approximation for the estimation of *S*_ini_, we also performed the numerical simulation with higher initial temperatures *T*_ini_=110, 125 nK (Supplementary Note 8 and Supplementary Fig. 5). More comprehensive study including lower *T*_ini_ can be found in Supplementary Fig. 8. The numerical results at higher *T*_ini_ show a distinct structure resulting from the incoherent NF contributions. As shown in Fig. 4a–c,d–f, thermal fluctuations destroy the SF and increase the NF, and the discrete structure are more visible. The experimental results with *V*_L_=7*E*_r_ and 10*E*_r_ are well explained with the numerical results with *T*_ini_=95 and 110 nK, respectively. In the deep lattice as shown in Fig. 4g–i (*V*_L_=13*E*_r_), the higher temperatures modify the relative height of the discrete peaks, and the experimental result is well explained by the results calculated at *T*_ini_=125 nK. The heating behaviour is consistent with the visibility measurement in the matter-wave interference, which also indicates heating due to the atom loading sequence (Supplementary Fig. 3 and Supplementary Note 4).

We finally discuss the noticeable deviation of the peak positions of multi-occupancies at deep lattices (for example, see Fig. 4g–i at around *ν*=−30 kHz). As regards the *m*-atom-occupancy in our simulation, we simply assume that a peak position is determined from (*m*−1)*δU*_0_. A more elaborate calculation that considers the interaction-broadening of the Wannier function, where the interaction strengths depend on *m* as *δU*_0_(*m*), is necessary to accurately predict the peak position.

## Discussion

We have employed a laser spectroscopy technique for ultracold bosonic atoms in a 3D optical lattice and performed a numerical analysis based on the formally derived sum rule relations. By using the very different interaction energies in the different electronic states, our spectroscopy technique provides outstanding resolution from the weakly to the strongly interacting regimes, and the observed spectra exhibit great changes in their shapes. Our numerical method enables us to calculate the excitation spectrum in finite temperature as a steady-state solution in the weak excitation regime, and effectively deals with the effect of the finite probe time in the experiment as a laser linewidth. The combination of the experimental and numerical study gives us a quantitative understanding of the coexistence of the SF and insulating states. Our approach paves the way to an exploration of the finite temperature Bose–Hubbard phase diagram including the quantum critical point, and can be applied to other types of the quantum many-body system, such as fermionic or Bose–Fermi mixtures in the optical lattice.

## Methods

### Preparation of ultracold Yb atoms in an optical lattice

We first prepare laser-cooled ^174^Yb atoms in a crossed optical dipole trap^26^. The wavelength of the dipole trap laser is 532 nm. After evaporative cooling, we obtain a Bose-Einstein condensate (BEC) of ^174^Yb, and load the BEC into a 3D optical lattice with a simple cubic geometry and a lattice constant of 266 nm by ramping up the optical lattice laser intensity. The lattice potential is linearly increased to a desired depth at a ramping rate of 15^−1^ *E*_r_/ms. The lattice potential is superimposed with a harmonic confinement with a trapping frequency of 140 Hz at 15*E*_r_. The intensity of the lattice laser is precisely controlled by the feedback of a monitored laser power. The lattice depth is calibrated by observing the diffraction of the trapped atoms induced by a pulsed lattice potential. In all of the spectroscopy experiments shown in Fig. 2, we fix the number of atoms *N* at ∼2.2(3) × 10^4^, the BEC transition temperature *T*_c_ at ∼160 nK, and the initial atom temperature *T*_ini_ at ∼95 nK.

### Spectroscopy method

To achieve the weak excitation of the atoms, we measure the number of excited atoms by using the repumping method. The ground-state atoms are directly excited to the ^3^P_2_(*m*=2) state by using a laser pulse with a wavelength of 507 nm and a pulse duration of 1 ms, where *m* denotes the magnetic quantum number. After the excitation pulse, we remove the ground-state atoms by using a blasting pulse that is resonant to the strong electric dipole ^1^S_0_↔^1^P_1_ transition (399 nm). Then, we repump the atoms in the excited state by using two repumping transitions, namely the ^3^P_0_↔^3^S_1_ transition (649 nm) and the ^3^P_2_↔^3^S_1_ transition (770 nm). The pulse durations for the blasting and the repumping are 0.2 and 0.5 ms, respectively. To detect the repumped atoms with a high signal-to-noise ratio, we recapture the atoms by using a magneto-optical trap (MOT) with the electric dipole ^1^S_0_↔^1^P_1_ transition, and measure the fluorescence from the MOT with an electron-multiplying charge-coupled-device camera with an exposure time of 100 ms. Although the atoms in the ^3^P_2_(*m*=2) state suffer from a Zeeman-sublevel-changing collision (Supplementary Note 6) and acquire kinetic energy during the inelastic collision, the MOT can capture the repumped atoms thanks to the large capture volume and the strong trapping force.

### Explicit formulae of sum rule relations

The sum rule imposes the condition that the first-order moment 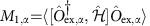 should be expressed by a combination of various thermal quantities or correlation functions for the atoms in the ground state before excitation:


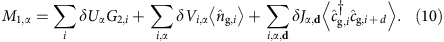


The second-order moment 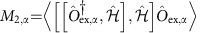 is also expressed as follows:


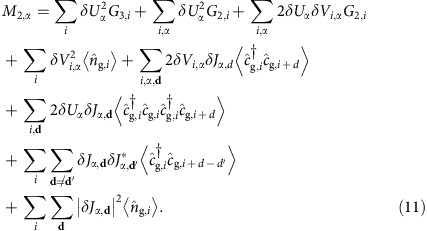


It is noted that the sixth term represents correlated hopping. The first-order sum rule provides information about a spectral peak position, which is discussed in refs 24, 29, and the second-order sum rule indicates the variance of the spectra. The comparison between the moments calculated from numerical and theoretical results is discussed in Supplementary Fig. 9 and Supplementary Note 12.

### Hubbard parameters

The Hubbard parameters, namely the hopping matrix *J*_g(e)_, the trapping potential *V*_g(e),*i*_ and the interaction strength *U*_gg,(ge)_ and the excitation probability *η*_*α*_, are determined in an *ab initio* manner by numerically calculating the Bloch and Wannier orbitals. We include the difference in the polarizability of the ^1^S_0_ and ^3^P_2_ states in the calculation. In addition, we consider the formation of two-body bound states consisting of the ^1^S_0_ and ^3^P_2_ atoms induced by the confinement of the lattice potential^33^, which causes the renormalization of the interaction strength *U*_ge_. By using the *ab initio* Bloch waves, we determine the two-body bound states^33^. This bound state formation only occurs when the energy scale of the onsite interaction becomes comparable to the Hubbard band gap^27^,^33^,^34^. We neglect other types of interaction renormalization, such as self-trapping^35^, because the number of atoms is small. The temperature in the lattice is determined on the assumption that the atom loading process is adiabatic. The thermal equilibrium temperature in the lattice, *T*_lat_, depends on *V*_L_.

## Additional information

**How to cite this article:** Kato, S. *et al*. Laser spectroscopic probing of coexisting superfluid and insulating states of an atomic Bose–Hubbard system. *Nat. Commun.* 7:11341 doi: 10.1038/ncomms11341 (2016).

## Supplementary Material

Supplementary Information Supplementary Figures 1-9, Supplementary Notes 1-12 and Supplementary References

## References

[b1] Jaksch D. & Zoller P. The cold atom Hubbard toolbox. Ann. Phys. 315, 52–79 (2005).

[b2] Bloch I., Dalibard J. & Zwerger W. Many-body physics with ultracold gases. Rev. Mod. Phys. 80, 885–964 (2008).

[b3] Greiner M., Mandel O., Esslinger T., Hänsch T. W. & Bloch I. Quantum phase transition from a superfluid to a Mott insulator in a gas of ultracold atoms. Nature 415, 39–44 (2002). 11780110 10.1038/415039a

[b4] Gerbier F. . Phase coherence of an atomic mott insulator. Phys. Rev. Lett. 95, 050404 (2005). 16090855 10.1103/PhysRevLett.95.050404

[b5] Trotzky S. . Suppression of the critical temperature for superfluidity near the Mott transition. Nat. Phys. 6, 998–1004 (2010).

[b6] Fölling S. . Spatial quantum noise interferometry in expanding ultracold atom clouds. Nature 434, 481–484 (2005). 15791249 10.1038/nature03500

[b7] Spielman I., Phillips W. & Porto J. Mott-Insulator Transition in a Two-Dimensional Atomic Bose Gas. Phys. Rev. Lett. 98, 080404 (2007). 17359074 10.1103/PhysRevLett.98.080404

[b8] Gemelke N., Zhang X., Hung C.-L. & Chin C. *In situ* observation of incompressible Mott-insulating domains in ultracold atomic gases. Nature 460, 995–998 (2009). 19693080 10.1038/nature08244

[b9] Bakr W. S. . Probing the superfluid-to-Mott insulator transition at the single-atom level. Science (New York, NY) 329, 547–550 (2010). 20558666 10.1126/science.1192368

[b10] Sherson J. F. . Single-atom-resolved fluorescence imaging of an atomic Mott insulator. Nature 467, 68–72 (2010). 20720540 10.1038/nature09378

[b11] Clément D., Fabbri N., Fallani L., Fort C. & Inguscio M. Exploring correlated 1D Bose gases from the superfluid to the Mott-insulator state by inelastic light scattering. Phys. Rev. Lett. 102, 155301 (2009). 19518645 10.1103/PhysRevLett.102.155301

[b12] Ernst P. T. . Probing superfluids in optical lattices by momentum-resolved Bragg spectroscopy. Nat. Phys. 6, 56–61 (2009).

[b13] Du X. . Bragg spectroscopy of a superfluid Bose—Hubbard gas. N. J. Phys. 12, 083025 (2010).

[b14] Stöferle T., Moritz H., Schori C., Köhl M. & Esslinger T. Transition from a Strongly Interacting 1D Superfluid to a Mott Insulator. Phys. Rev. Lett. 92, 130403 (2004). 15089587 10.1103/PhysRevLett.92.130403

[b15] Schori C., Stöferle T., Moritz H., Köhl M. & Esslinger T. Excitations of a superfluid in a three-dimensional optical lattice. Phys. Rev. Lett. 93, 240402 (2004). 15697784 10.1103/PhysRevLett.93.240402

[b16] Mark M. J. . Precision measurements on a tunable Mott insulator of ultracold atoms. Phys. Rev. Lett. 107, 175301 (2011). 22107531 10.1103/PhysRevLett.107.175301

[b17] Gerbier F., Fölling S., Widera A., Mandel O. & Bloch I. Probing number squeezing of ultracold atoms across the superfluid-Mott insulator transition. Phys. Rev. Lett. 96, 090401 (2006). 16606244 10.1103/PhysRevLett.96.090401

[b18] Campbell G. K. . Imaging the Mott insulator shells by using atomic clock shifts. Science (New York, NY) 313, 649–652 (2006). 16888134 10.1126/science.1130365

[b19] Cappellini G. . Direct observation of coherent interorbital spin-exchange dynamics. Phys. Rev. Lett. 113, 120402 (2014). 25279608 10.1103/PhysRevLett.113.120402

[b20] Zhang X. . Spectroscopic observation of SU(N)-symmetric interactions in Sr orbital magnetism. Science 345, 1467–1473 (2014). 25147278 10.1126/science.1254978

[b21] Scazza F. . Observation of two-orbital spin-exchange interactions with ultracold SU(N)- symmetric fermions. Nat. Phys. 10, 779–784 (2014).

[b22] Inaba K. & Yamashita M. Theoretical analysis on spectroscopy of atomic Bose-Hubbard Systems. Preprint at http://arxiv.org/abs/1507.06399 (2015).

[b23] Oktel M., Killian T. C., Kleppner D. & Levitov L. Sum rule for the optical spectrum of a trapped gas. Phys. Rev. A 65, 033617 (2002).

[b24] Hazzard K. R. A. & Mueller E. J. Hyperfine spectra of trapped bosons in optical lattices. Phys. Rev. A 76, 063612 (2007).

[b25] Yamaguchi A., Uetake S., Kato S., Ito H. & Takahashi Y. High-resolution laser spectroscopy of a Bose—Einstein condensate using the ultranarrow magnetic quadrupole transition. N. J. Phys. 12, 103001 (2010).

[b26] Kato S., Shibata K., Yamamoto R., Yoshikawa Y. & Takahashi Y. Optical magnetic resonance imaging with an ultra-narrow optical transition. Appl. Phys. B 108, 31–38 (2012).

[b27] Kato S., Sugawa S., Shibata K., Yamamoto R. & Takahashi Y. Control of resonant interaction between electronic ground and excited states. Phys. Rev. Lett. 110, 173201 (2013). 23679722 10.1103/PhysRevLett.110.173201

[b28] Kato S. . Observation of long-lived van der Waals molecules in an optical lattice. Phys. Rev. A 86, 043411 (2012).

[b29] Hazzard K. R. A. & Mueller E. J. Many-body physics in the radio-frequency spectrum of lattice bosons. Phys. Rev. A 81, 033404 (2010).

[b30] Fukuhara T., Sugawa S., Sugimoto M., Taie S. & Takahashi Y. Mott insulator of ultracold alkaline-earth-metal-like atoms. Phys. Rev. A 79, 041604 (2009).

[b31] Ohashi Y., Kitaura M. & Matsumoto H. Itinerant-localized dual character of a strongly correlated superfluid Bose gas in an optical lattice. Phys. Rev. A 73, 033617 (2006).

[b32] Sun K., Lannert C. & Vishveshwara S. Probing condensate order in deep optical lattices. Phys. Rev. A 79, 043422 (2009).

[b33] Büchler H. P. Microscopic derivation of Hubbard parameters for cold atomic gases. Phys. Rev. Lett. 104, 090402 (2010). 20366972 10.1103/PhysRevLett.104.090402

[b34] Busch T., Englert B.-G., Rzażewski K. & Wilkens M. Two cold atoms in a harmonic trap. Found. Phys. 28, 549–559 (1998).

[b35] Lühmann D.-S., Bongs K., Sengstock K. & Pfannkuche D. Self-trapping of Bosons and Fermions in optical lattices. Phys. Rev. Lett. 101, 050402 (2008). 18764376 10.1103/PhysRevLett.101.050402

[b36] Capogrosso-Sansone B., Prokof'ev N. V. & Svistunov B. V. Phase diagram and thermodynamics of the three-dimensional Bose-Hubbard model. Phys. Rev. B 75, 134302 (2007).

