# Supplementary material for: Laser spectroscopic probing of coexisting superfluid and insulating states of an atomic Bose–Hubbard system
Source: Nat Commun. 2016 Apr 20;7:11341. doi: 10.1038/ncomms11341 (PMC4843003; doi:10.1038/ncomms11341)
Supplement: Supplementary Information — Supplementary Figures 1-9, Supplementary Notes 1-12 and Supplementary References [file ncomms11341-s1.pdf]

SUPPLEMENTARY FIGURES

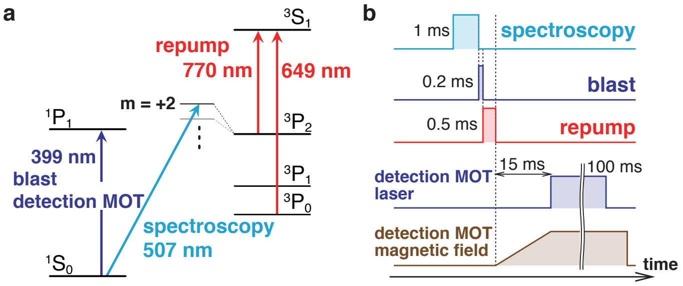

Supplementary Figure 1. **Energy diagram and pulse sequence of the spectroscopy technique.** **a** Energy levels of an ytterbium atom relevant to the experiment. **b** Spectroscopy pulse sequence.

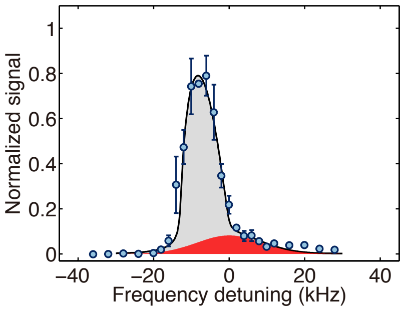

Supplementary Figure 2. **Excitation spectrum of the BEC in the optical trap.** Circles denote the experimental observations and error bars indicate s.e.m. The normalization procedure of the spectrum amplitude is the same as used in Fig. 2 in the main text. The black solid line shows the calculated spectrum of the sum of the contributions from the condensed and thermal components. The calculated spectrum is convoluted with a Lorentzian function with a linewidth of 1 kHz to consider the linewidth of the excitation laser. The red shaded area indicates the contribution of the thermal component.

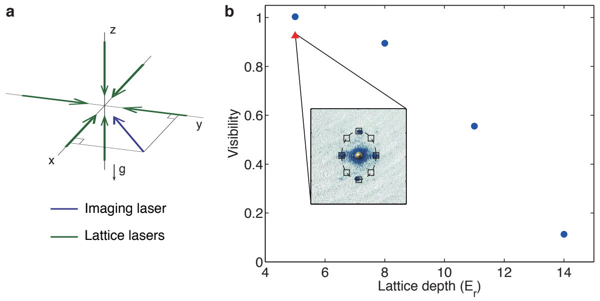

Supplementary Figure 3. **Visibility of the interference fringe.** **a** Schematic of the absorption imaging set-up. **b** Visibility of the matter-wave interference and its recovery by ramping down the lattice potential. The blue circles show the visibility as a function of the final lattice depth. The red triangle shows the visibility, for which the lattice depth was decreased to $5E_r$ in 20 ms after ramping up to 14 E_r. The inset shows the absorption image, and the boxed regions are used for the visibility calculation.

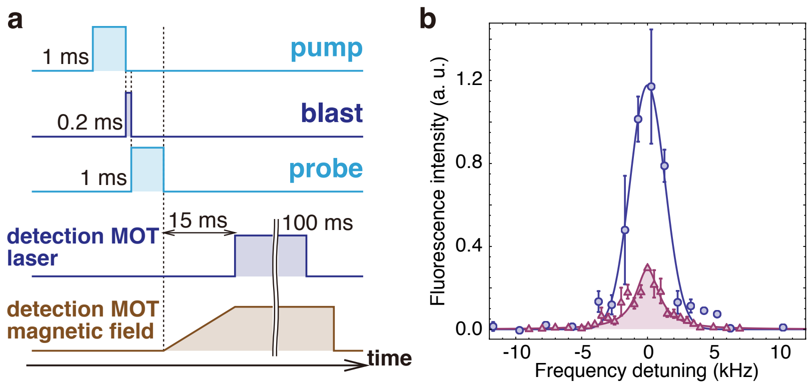

Supplementary Figure 4. **Inhomogeneous broadening in the spectrum.** **a** Experimental pulse sequence for the pump-and-probe method. **b** An excitation spectrum for the singly-occupied sites with inhomogeneous broadening (circles) and a hole-burning like spectrum (triangles). The circles and triangles are plotted as a function of the excitation laser frequency and the frequency detuning of the probe laser from the pump laser (see Supplementary Note 5), respectively. The pump laser frequency is fixed at the peak of the resonance, which corresponds to zero frequency in the plot. The lattice depth is $15\mathrm{E_r}$. Error bars indicate s.e.m. The solid lines denote a Gaussian fit and a Lorentzian fit to the circles and the triangles, respectively. The FWHMs for the Gaussian and the Lorentzian functions are 3.1 and 2.1 kHz, respectively.

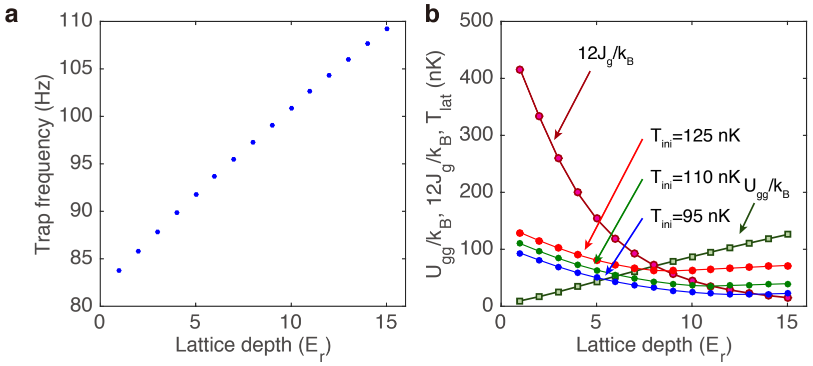

Supplementary Figure 5. **Trapping frequencies and temperature in the lattice.** **a** Geometric mean of the trap frequency as a function of the lattice depth. **b** $U_{\mathrm{gg}}/k_{\mathrm{B}}$, $J_{\mathrm{g}}/k_{\mathrm{B}}$ and T_{lat} as a function of the lattice depth.

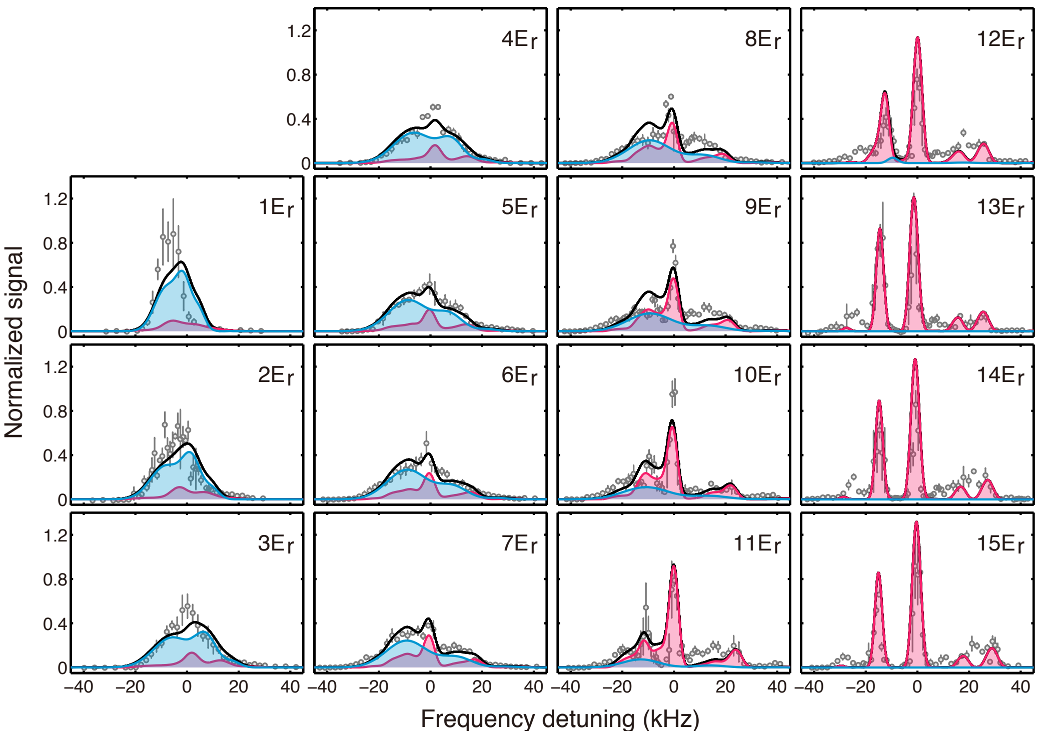

Supplementary Figure 6. **Superfluid and normal fluid contributions in the spectra.** We assume an initial atom temperature of 95 nK for the calculation. The blue and red shaded areas denote the contributions made by the superfluid and the normal fluid to the numerically calculated spectra (black solid lines). The grey circles and error bars denote the experimental observations, which are the same as the data in Fig. 2 in the main text.

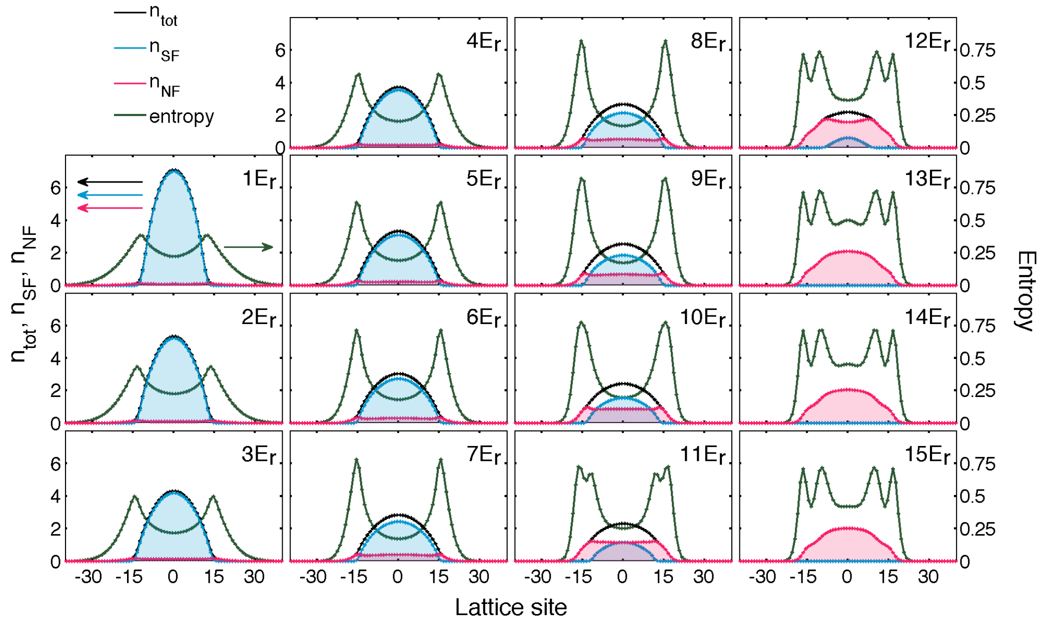

Supplementary Figure 7. **Calculated atom distributions and entropies in the optical lattices at various lattice depths.** We assume the initial atom temperature is 95 nK for calculation. The blue and red shaded areas correspond to the SF and NF components and the black solid lines denote their sum. The green solid lines denote the entropies.

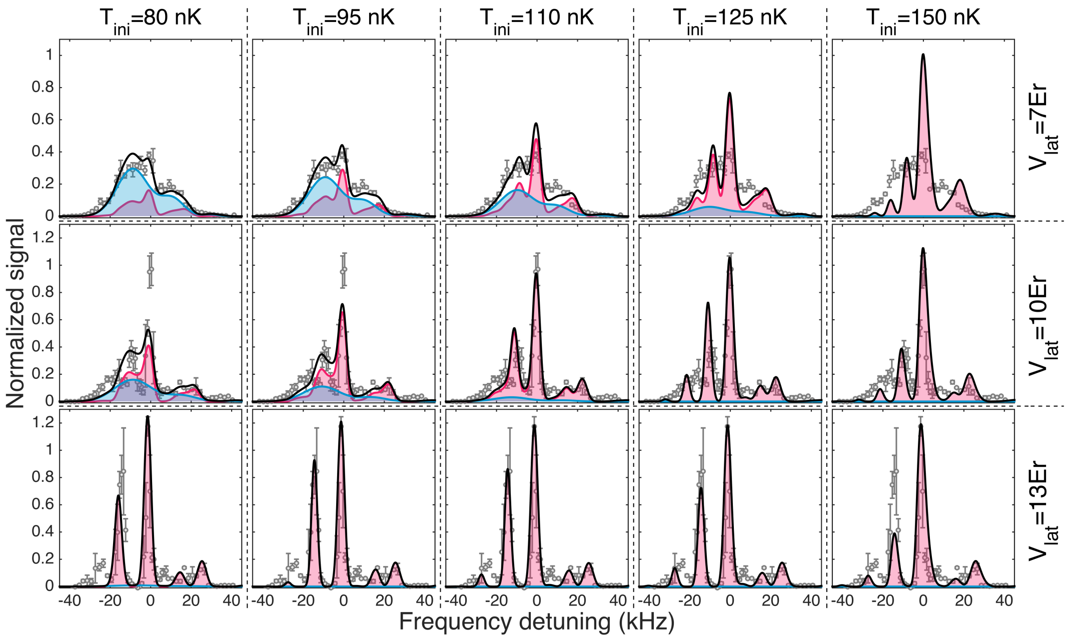

Supplementary Figure 8. **Superfluid and normal fluid contributions with different initial temperatures.** We assume the initial atom temperature to be 80, 95, 110, 125, 150 nK for the calculations. The color scheme is the same as in Supplementary Fig. 6.

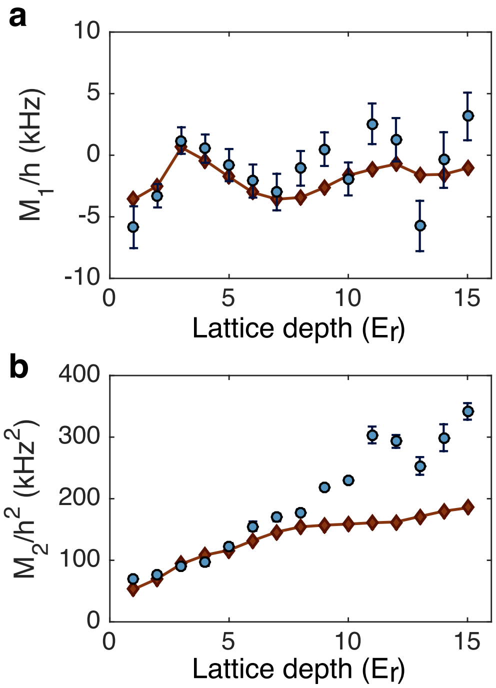

Supplementary Figure 9. **Spectral moments derived from the excitation spectra.** Spectral moments M_1 (**a**) and M_2 (**b**) as functions of the lattice depth. The circles and diamonds show the results calculated using the experimental observations and the numerical simulations shown in Fig. 2 in the main text, respectively. Error bars denote s.d.

SUPPLEMENTARY NOTES

Supplementary Note 1: Experimental spectroscopy procedure

Supplementary Fig. 1**a** shows an energy diagram relevant to the experiment, and Supplementary Fig. 1**b** is a diagram of the spectroscopy pulse sequence. The procedure for preparing cold Yb atoms and the spectroscopy method are discussed in Methods in the main text and the details are described in Ref. [1].

Supplementary Note 2: Separately induced Rabi oscillations in different fillings

The experimental method that we used to observe the Rabi oscillations shown in Fig. 1**d** in the main text is as follows. To determine the resonance frequencies for each filling, we first measure the excitation spectrum with a lattice depth of $22\mathrm{E_r}$, as shown in Fig. 1**c**. We use the $^3\mathrm{P}_2(m=0)$ state as the excited state. We then change the excitation pulse duration and measure the number of atoms in the excited state with the excitation laser frequency fixed at each peak position. The intensity of the laser is fixed in all the measurements in Fig. 1**d**. To measure the number of excited atoms, we perform a blast and repumping procedure in the same manner as in the other spectroscopy experiments, and measure the repumped atoms by using the standard absorption imaging method with the $^1\mathrm{S}_0-{}^1\mathrm{P}_1$ transition. The number of atoms in the trap is 7.8×10^4 for the observations with the singly and the doubly occupied sites and 1.1×10^5 for the observation with the triply occupied sites in Fig. 1**d**.

Supplementary Note 3: Spectroscopy of the Yb BEC in the optical trap

We perform the spectroscopy of the Yb BEC in the optical trap without the lattice potential (V_L=0), and observe a density-dependent mean-field shift and broadening [2, 3] as shown in Supplementary Fig. 2. The origin of the frequency detuning is the same as the frequency origin in Fig. 2 in the main text. The spectrum is understood as the sum of the contributions from the condensed and thermal components. Their ratio is consistent with a temperature of 95 nK evaluated from the spectrum width of the thermal component and independently performed time-of-flight temperature measurements.

Supplementary Note 4: Checking adiabaticity with visibility measurements of the matter wave interference

To check the adiabaticity during the lattice ramp up, we measure the visibility of the matter wave interference and its recovery after the ramp down of the lattice potential. The laser setup is shown schematically in Supplementary Fig. 3**a**, which is the same configuration in the main text. We utilize the $^1\mathrm{S}_0\leftrightarrow{}^1\mathrm{P}_1$ transition for the imaging. The absorption images are taken after a ballistic expansion of 14 ms. In these measurements, the lattice ramp up rate is $0.1\mathrm{E_r}$/ms. The reduction in visibility (the blue circles in Supplementary Fig. 3**b**) and its recovery (the red triangle in Supplementary Fig. 3**b**) indicate that the lattice loading does not induce excess heating.

Supplementary Note 5: Inhomogeneous broadening caused by the optical trap

We investigate the origin of the width of the excitation spectrum of the singly occupied sites in the deep lattice potential. The spectral width is contributed to by the inhomogeneous broadening caused by different Stark shifts between the $^1\mathrm{S}_0$ and the $^3\mathrm{P}_2\,(\mathrm{m}=2)$ states with the trapping lasers. This can be confirmed by the hole-burning spectroscopy measurement described below. Here, we use the pump-and-probe method as shown in the pulse sequence in Supplementary Fig. 4**a**. First, we employ an excitation pump pulse ($^1\mathrm{S}_0\leftrightarrow{}^3\mathrm{P}_2\,(\mathrm{m}{=}2)$) at a certain frequency, and then atoms remaining in the ground state are removed by a blast laser pulse. Next, we return the atoms in the $^3\mathrm{P}_2\,(\mathrm{m}=2)$ state to the $^1\mathrm{S}_0$ state by employing another laser pulse, the frequency of which is scanned around the first pump pulse frequency. Finally, we observe the number of returned atoms by the fluorescence measurement. Note that this procedure allows us to distinguish between the homogeneous and inhomogeneous broadenings of the spectra. Supplementary Fig. 4**b** shows the spectra obtained at lattice depths of $15\mathrm{E_r}$. The observed narrower spectrum gives a spectral width of about 2.1 kHz, which is mainly limited by our laser linewidth. It should be noted that the pump and probe laser pulses are taken from the same laser source with an acousto-optic deflector driven by two RF sources

with stable frequencies. This inhomogeneous broadening effect is properly incorporated in our numerical calculations of the spectra by taking account of the differential light shifts, namely $V_{\mathrm{g},i}$ and $V_{\mathrm{e},i}$.

Supplementary Note 6: Inelastic collisions in the $^3\mathrm{P}_2$ in the spectroscopy

It should be noted that the atom number measurement in the present method is insensitive to the two-body Zeeman-sublevel-changing collision (ZSLCC) observed in the previous study [4]. Although the estimated decay time caused by the ZSLCC for the $^3\mathrm{P}_2$ (m = 2) atoms in the doubly occupied site is about 1 ms, which is almost the same as the excitation pulse duration, the kinetic energy released by the ZSLCC is expected to be about 11 μK per atom at a magnetic field of 220 mG in this case. Our numerical calculation shows that the kinetic energy released by the ZSLCC is insufficient to escape from the detection MOT capture range of 1.5 mm in 16.7 ms before starting the MOT for the detection. In fact, the behavior of the fluorescence intensity signal as a function of the hold time before the application of the repumping pulse is consistent with our simulation. It is also true that a measurement performed in a different experimental set-up with a reduced capture range shows a reduced signal for the doubly and triply occupied sites.

Supplementary Note 7: Finite temperature Gutzwiller approximation

To calculate the excitation spectrum, we require the thermodynamic quantities that appear in the explicit form of $M_{\ell,\alpha}$ in Eqs. (10) and (11) in Methods. We use the the Gutzwiller type approximation[5], which enables us to effectively analyze the thermal equilibrium properties of the Hubbard Hamiltonian in Eq. (1) in the main text. This approximation is a mean field approximation that considers up to the first order correction in terms of J_g, and then the Hamiltonian $\hat{\mathcal{H}}_\mathrm{g}$ is approximated by a set consisting of the effective local Hamiltonian $\hat{\mathcal{H}}_\mathrm{loc}$;

$$\hat{\mathcal{H}}_\mathrm{g} = \sum_i \hat{\mathcal{H}}_\mathrm{loc},$$
$$\hat{\mathcal{H}}_\mathrm{loc} = \tilde{J}_{\mathrm{g},i}\hat{c}^\dagger_{\mathrm{g},i} + \tilde{J}^*_{\mathrm{g},i}\hat{c}_{\mathrm{g},i} + V_i\hat{n}_{\mathrm{g},i} + U_\mathrm{gg}\frac{\hat{n}_{\mathrm{g},i}(\hat{n}_{\mathrm{g},i}-1)}{2}, \tag{1}$$

where $\tilde{J}_{\mathrm{g},i}$ is self-consistently calculated by the condition $\tilde{J}_{\mathrm{g},i} = -\sum_\mathbf{d} J_\mathrm{g}\langle\hat{c}_{\mathrm{g},i+d}\rangle$. A finite $\langle\hat{c}_{\mathrm{g},i}\rangle = c_{\mathrm{g},i}$ effectively describes the Bose-Einstein condensates within the mean-field approximation. The number of condensed SF atoms is given by $n_{\mathrm{SF},i} = |c_{\mathrm{g},i}|^2$. The creation (annihilation) operator of NF atoms at the ith site is effectively given by $\hat{c}^\dagger_{\mathrm{NF},i} = \hat{c}^\dagger_{\mathrm{g},i} - c^*_{\mathrm{g},i}$ ($\hat{c}_{\mathrm{NF},i} = \hat{c}_{\mathrm{g},i} - c_i$), where the NF atoms always satisfy the condition $\langle\hat{c}_{\mathrm{NF},i}\rangle = 0$ and the number of NF atoms is defined as $n_{\mathrm{NF},i} = \langle\hat{c}^\dagger_{\mathrm{NF},i}\hat{c}_{\mathrm{NF},i}\rangle$. The actual number of atoms $n_{g,i} = \langle\hat{c}^\dagger_{\mathrm{g},i}\hat{c}_{\mathrm{g},i}\rangle$ is now given by the sum of the SF and NF atoms: $n_{g,i} = n_{\mathrm{SF},i} + n_{\mathrm{NF},i}$. Within the above local approximation, the long-range correlations are given by $\langle\hat{c}^\dagger_{\mathrm{g},i}\hat{c}_{\mathrm{g},j}\rangle \sim c^*_{\mathrm{g},i}c_{\mathrm{g},j}$, where the long-range correlations of the NF atoms are approximately neglected $\langle\hat{c}^\dagger_{\mathrm{NF},i}\hat{c}_{\mathrm{NF},j}\rangle = 0$. We should note that the NF states connect adiabatically to the MI state at low temperatures. For a large $U_\mathrm{gg}(\gg J_\mathrm{g})$ at zero temperature, the ground state of $\hat{\mathcal{H}}_\mathrm{loc}$ is given by the m-atom number states with an energy $E_m = m(m-1)U_\mathrm{gg}/2 + V_i m$, where the entropy is surely zero. As the temperature increases, other number states are mixed, and then the entropy increases. By calculating the entropy per site, we can thus effectively evaluate the MI-NF crossover within this local approximation (see Supplementary Fig. 7).

Supplementary Note 8: Calculation of thermodynamic quantities

We calculate the thermal states at a certain temperature T_lat using an exact diagonalization method by appropriately eliminating the states with a large number of atoms. In the calculation, we use the actual experimental parameters, such as the atom number, temperature, and inhomogeneous trapping potential. We plot the trapping frequency for the electronic ground state atoms as a function of the lattice depth in Supplementary Fig. 5**a**. We take account of the anharmonicity of the trapping potential resulting from the Gauss shapes of the lasers with a waist size of about 50 μm.

A temperature T_lat is estimated numerically under the assumption of adiabatic atom loading; hence the process is assumed to be isentropic. The initial entropy of the atomic gases without lattice potential is given as follows by neglecting the interactions between trapped atoms (assuming the ideal Bose gas): $S_\mathrm{ini} \sim 3.6N_\mathrm{tot}(T_\mathrm{ini}/T_c)^3$, where

$T_c(\sim 160\,\mathrm{nK})$ is the BEC transition temperatures of the initial atomic gas. Note that we consider effectively the non-adiabatic heating effects and also the error from the ideal Bose gas assumption for estimation of S_{ini} by taking account of the uncertainty of the initial temperatures $T_{\mathrm{ini}} = 95$ nK $+\delta T$. As mentioned above, the initial temperature without lattice ramping of 95 nK is determined from the spectrum and time-of-flight imaging (see Supplementary Fig. 2). Nonadiabatic heating effects during lattice ramping are also estimated with the visibility measurement. We found that the coherent peak structures in the time-of-flight images are reproduced even after we ramp lattice up and down. It suggests that the heating effects are not significant and $\delta T \ll 65$ nK ($\sim T_c - 95$ nK). To further consider the temperature uncertainty, we adopt three different initial temperatures (T_{ini}=95, 110, 125 nK) for the calculations in Fig. 4 in the main text. From Fig. 4, we estimated the non-adiabatic heating δT as $\delta T \lesssim 30$ nK. We plot calculated T_{lat} as a function of the lattice depth V_L with the initial temperatures in Supplementary Fig. 5**b**. In addition, Supplementary Fig. 5**b** also shows $U_{\mathrm{gg}}/k_{\mathrm{B}}$ and $12J_{\mathrm{g}}/k_{\mathrm{B}}$ for comparison.

Supplementary Note 9: Binary fluid approximation and spectrum calculation

Here, we explain the binary fluid approximation, which allows us to calculate the finite temperature spectra in inhomogeneous systems numerically. The same method has been described elsewhere [6], where it is mainly applied to RF spectroscopy. We explain it briefly to make the present paper self-contained.

The binary fluid approximation assumes that $I_\alpha(\nu)$ can be decomposed into two components consisting of SF and NF atoms:

$$I_\alpha(\nu) = I_{\mathrm{SF},\alpha}(\nu) + I_{\mathrm{NF},\alpha}(\nu). \tag{2}$$

The SF atoms have definite phase coherence and condense into the lowest eigenstate that spreads over the several lattice sites. In contrast, the NF atoms have no phase coherence and correspond to the localized number states on condition that $J_{\mathrm{g}} \ll U_{\mathrm{gg}}$ and $J_{\mathrm{g}} \ll k_{\mathrm{B}}T_{\mathrm{lat}}$. We deal with the interactions between the macroscopically condensed SF atoms and the distinguishable NF atoms in a mean-field manner so that we can obtain the dynamical quantities $I_{\mathrm{SF},\alpha}(\nu)$ and $I_{\mathrm{NF},\alpha}(\nu)$ separately.

To properly calculate the two components of the spectra, $I_{\mathrm{SF},\alpha}(\nu)$ and $I_{\mathrm{NF},\alpha}(\nu)$, we consider the spectral moments

$$M_{\ell,\alpha} = \int d\nu (h\nu)^\ell I_{\mathrm{NF},\alpha}(\nu) + \int d\nu (h\nu)^\ell I_{\mathrm{SF},\alpha}(\nu). \tag{3}$$

We use these formulae up to the second order i.e., $\ell = 0, 1$, and 2. The zero-th order sum rule simply offers the condition associated with the total number of atoms: $n_{\mathrm{tot}} = n_{\mathrm{NF}} + n_{\mathrm{SF}}$, which is always satisfied within the Gutzwiller approximation. On the other hand, the first and second order sum rules provide the mean value of the excitation energy and its variance. We discuss the properties of $I_{\mathrm{NF},\alpha}(\nu)$ and $I_{\mathrm{SF},\alpha}(\nu)$ separately, and then we obtain Eqs. (8) and (9) in the main text.

Spectra of uncondensed normal-state atoms

Here we begin with $I_{\mathrm{NF},\alpha}(\nu)$. As a first step, it is easy to consider a simple case where all the atoms prior to spectral excitation are in the local state as an uncondensed state so that $n_{\mathrm{SF},i} = 0$ and $\langle \hat{c}^\dagger_{\mathrm{g},i}\hat{c}_{\mathrm{g},i+d}\rangle \sim 0$, and accordingly $I_{\mathrm{SF},\alpha}(\nu)$ vanishes. The density matrix of the localized state at finite temperatures is given by $\prod_i(\sum_m e^{-E_{m,i}/(k_{\mathrm{B}}T_{\mathrm{lat}})}|m\rangle_{ii}\langle m|)/Z_i$, where

$$E_{m,i} = \frac{m(m-1)}{2}U_{\mathrm{gg}} + mV_{\mathrm{g},i}$$

is the energy of the local number state $|m\rangle_i$, and Z_i is the partition function for the i-th site. The spectra can be obtained in the form of the Lehman representation:

$$I_{\mathrm{NF},\alpha}(\nu) = \sum_{i,m} W_{\mathrm{NF},i\alpha m}\ \delta(\nu - p_{\mathrm{NF},i\alpha m}). \tag{4}$$

The spectral weight $W_{\mathrm{NF},i\alpha m}$ and the peak position $p_{\mathrm{NF},i\alpha m}$ are given by

$$W_{\mathrm{NF},i\alpha m} = |{}_i\langle m-1;1_\alpha|\hat{O}_{\mathrm{ex},\alpha}|m\rangle_i|^2 e^{-\frac{E_{m,i}-\Omega_i}{k_{\mathrm{B}}T_{\mathrm{lat}}}},$$
$$hp_{\mathrm{NF},i\alpha m} = E'_{m-1;1_\alpha,i} - E_{m,i},$$

where $|m-1;1_\alpha\rangle_i = \hat{c}^\dagger_{\mathrm{e},i\alpha}\hat{c}_{\mathrm{g},i}|m\rangle_i$ represents the number state consisting of both $(m-1)$ unexcited atoms and a single excited atom at the i-th site.

$$E'_{m;1_\alpha,i} = mU_{\mathrm{ge},\alpha} + V_{\mathrm{e},i} + \Delta_\alpha + E_{m,i}$$

is the corresponding energy, and $\Omega_i (= -k_\mathrm{B}T_\mathrm{lat}\ln\sum_m e^{-E_{m,i}/(k_\mathrm{B}T_\mathrm{lat})})$ is the grand potential at the i-th site. The spectral peak position $p_{\mathrm{NF},i\alpha m}$ reduces to

$$hp_{\mathrm{NF},i\alpha m} = E'_{m-1;1_\alpha,i} - E_{m,i} = (m-1)\delta U_\alpha + \delta V_{i,\alpha}. \tag{5}$$

By using the explicit form of $\hat{O}_{\mathrm{ex},\alpha}$, the peak weight $W_{\mathrm{NF},i\alpha m}$ reduces to

$$W_{\mathrm{NF},i\alpha m} = me^{-(E_{m,i}-\Omega_i)/(k_\mathrm{B}T_\mathrm{lat})}, \tag{6}$$

where $e^{-(E_{m,i}-\Omega_i)/(k_\mathrm{B}T_\mathrm{lat})}$ is the Boltzmann factor of the number state $|m\rangle_i$, and the factor m corresponds to the Bose enhancement factor. For a uniform system, it should be noted that the spectra at finite temperatures have multi-peak structures depending on the thermal distributions of the number states, while at zero temperature they become a single peak centered at $\delta U_\alpha(m-1) + \delta V_{i,\alpha}$, which is consistent with the previous study in Ref. [7]. Note that the multi-peak structure reflects the number fluctuations caused by the finite temperature effects: Namely, larger number fluctuations induce more peaks, while the MI state at zero temperature without fluctuations has a single peak.

The above expression Supplementary Eq. (4) of the spectra for NF is appropriate when the long-range correlation $\langle\hat{c}^\dagger_{\mathrm{g},i}\hat{c}_{\mathrm{g},j}\rangle$ is much smaller than the onsite correlation $\langle\hat{c}^\dagger_{\mathrm{g},i}\hat{c}_{\mathrm{g},i}\rangle$, because a local approximation is appropriate under this condition. When $J_\mathrm{g} = 0$, the condition of a negligible $\langle\hat{c}^\dagger_{\mathrm{g},i}\hat{c}_{\mathrm{g},j}\rangle$ is exactly satisfied: $\langle\hat{c}^\dagger_{\mathrm{g},i}\hat{c}_{\mathrm{g},j}\rangle = 0$. In addition, we consider that the expression also well describes the spectra for the Mott states ($J_\mathrm{g} \neq 0$ and $J_\mathrm{g} \ll k_\mathrm{B}T_\mathrm{lat} \ll U_\mathrm{gg}$), because the strong interaction U_gg supresses the long-range correlations. Furthermore, a large trapping potential $|V_{\mathrm{g},i} - V_{\mathrm{g},j}| \gg J_\mathrm{g}$ also supresses the long-range correlations.

Let us consider the sum rules by assuming a negligible $\langle\hat{c}^\dagger_{\mathrm{g},i}\hat{c}_{\mathrm{g},j}\rangle$. The spectral moment within the local approximation $M^{(\mathrm{loc})}_{1,\alpha}$ can be calculated as $\int h\nu I_{\mathrm{NF},\alpha}(\nu)d\nu = \sum_{i,m} W_{\mathrm{NF},i\alpha m}\, p_{\mathrm{NF},i\alpha m}$ using a local form of spectra in Supplementary Eq. (4). By substituting Supplementary Eqs. (5) and (6), and by using the following two equalities to derive the above form:

$$\langle\hat{c}^\dagger_{\mathrm{g},i}\hat{c}^\dagger_{\mathrm{g},i}\hat{c}_{\mathrm{g},i}\hat{c}_{\mathrm{g},i}\rangle = \sum_m m(m-1)e^{-(E_{m,i}-\Omega_i)/(k_\mathrm{B}T_\mathrm{lat})}, \tag{7}$$

$$\langle\hat{n}_{\mathrm{g},i}\rangle = \sum_m me^{-(E_{m,i}-\Omega_i)/(k_\mathrm{B}T_\mathrm{lat})},$$

we obtain

$$M^{(\mathrm{loc})}_{1,\alpha} = \sum_i \delta U_\alpha\langle\hat{c}^\dagger_{\mathrm{g},i}\hat{c}^\dagger_{\mathrm{g},i}\hat{c}_{\mathrm{g},i}\hat{c}_{\mathrm{g},i}\rangle + \sum_{i,\alpha}\delta V_{i,\alpha}\langle\hat{n}_{g,i}\rangle, \tag{8}$$

which is equivalent to $M_{1,\alpha}$ in Eq. (10) in the main text if $\langle\hat{c}^\dagger_{\mathrm{g},i}\hat{c}_{\mathrm{g},j}\rangle$ is negligible. Thus, the first order sum rule is properly described in Supplementary Eq. (4) within the local approximation.

In contrast, the second order sum rule is not straightforward. In the same way as above, by using the additional equality

$$\langle\hat{c}^\dagger_{\mathrm{g},i}\hat{c}^\dagger_{\mathrm{g},i}\hat{c}^\dagger_{\mathrm{g},i}\hat{c}_{\mathrm{g},i}\hat{c}_{\mathrm{g},i}\hat{c}_{\mathrm{g},i}\rangle = \sum_m m(m-1)(m-2)e^{-(E_{m,i}-\Omega_i)/(k_\mathrm{B}T_\mathrm{lat})},$$

we obtain the second moment within the local approximation

$$\begin{aligned} M^{(\mathrm{loc})}_{2,\alpha} = &\sum_i \delta U^2_\alpha\langle\hat{c}^\dagger_{\mathrm{g},i}\hat{c}^\dagger_{\mathrm{g},i}\hat{c}^\dagger_{\mathrm{g},i}\hat{c}_{\mathrm{g},i}\hat{c}_{\mathrm{g},i}\hat{c}_{\mathrm{g},i}\rangle + \sum_i \delta U^2_\alpha\langle\hat{c}^\dagger_{\mathrm{g},i}\hat{c}^\dagger_{\mathrm{g},i}\hat{c}_{\mathrm{g},i}\hat{c}_{\mathrm{g},i}\rangle \\ &+ \sum_i 2\delta U_\alpha\delta V_{i,\alpha}\langle\hat{c}^\dagger_{\mathrm{g},i}\hat{c}^\dagger_{\mathrm{g},i}\hat{c}_{\mathrm{g},i}\hat{c}_{\mathrm{g},i}\rangle + \sum_i \delta V^2_{i,\alpha}\langle\hat{n}_{\mathrm{g},i}\rangle. \end{aligned}$$

Even though the long-range correlations are negligible, $M^{(\mathrm{loc})}_{2,\alpha}$ is not equivalent to $M_{2,\alpha}$ in Eq. (11) in the main text, which includes an additional term $\sum_i\sum_\mathbf{d}|\delta J_{\alpha,\mathbf{d}}|^2\langle\hat{n}_{\mathrm{g},i}\rangle$. This additional term is the last term in Eq. (11). By comparing this term and the second to last term in Eq. (11) $\sum_i\sum_{\mathbf{d}\neq\mathbf{d}'}\delta J_{\alpha,\mathbf{d}}\delta J^*_{\alpha,\mathbf{d}'}\langle\hat{c}^\dagger_{\mathrm{g},i}\hat{c}_{\mathrm{g},i+d-d'}\rangle$, we find that the round-trip hopping process induces the onsite correlation $\langle\hat{n}_{\mathrm{g},i}\rangle$.

The discrepancy mentioned above can be resolved as follows. We replace the delta function in Supplementary Eq. (4) with a function with a finite spectral width, such as a Gaussian:

$$I_{\mathrm{NF},\alpha}(\nu) = \sum_{i,m} W_{\mathrm{NF},i\alpha m} \frac{\exp\Big(- h^2(\nu - p_{\mathrm{NF},i\alpha m})^2/(2\sigma^2_{\mathrm{NDS},\alpha})\Big)}{\sigma_{\mathrm{NDS},\alpha}\sqrt{2\pi}}, \tag{9}$$

where $\sigma_{\mathrm{NDS},\alpha}$ is the spectral width resulting from the hopping term $\sqrt{\sum_{\mathbf{d}} |\delta J_{\alpha,\mathbf{d}}|^2}$. The extended Lehman representation of Supplementary Eq. (9) properly satisfies the sum rules. The zero-th and first order sum rules are unchanged from the results obtained with the expression of Supplementary Eq. (4). The second order sum rule is extended as follows:

$$\begin{aligned}
&\int (h\nu)^2 \sum_{i,m} W_{\mathrm{NF},i\alpha m} \frac{e^{-h^2(\nu - p_{\mathrm{NF},i\alpha m})^2/2\sigma^2_{\mathrm{NDS},\alpha}}}{(\sigma_{\mathrm{NDS},\alpha}\sqrt{2\pi})} d\nu \\
&= \sum_{i,m} W_{\mathrm{NF},i\alpha m}(h^2 p^2_{\mathrm{NF},i\alpha m} + \sigma^2_{\mathrm{NDS},\alpha}) \\
&= \int (h\nu)^2 \sum_{i,m} W_{\mathrm{NF},i\alpha m}\ \delta(\nu - p_{\mathrm{NF},i\alpha m}) d\nu + \sum_{i,m} W_{\mathrm{NF},i\alpha m}\sigma^2_{\mathrm{NDS},\alpha}.
\end{aligned} \tag{10}$$

The additional term $\sum_{i,m} W_{\mathrm{NF},i\alpha m}\sigma^2_{\mathrm{NDS},\alpha} (= \sum_i \langle \hat{n}_{\mathrm{g},i}\rangle \sigma^2_{\mathrm{NDS},\alpha})$ can properly describe a finite spectral width resulting from the round-trip hopping. The above expression Supplementary Eq. (9) is already given as Eq. (8) in the main text. From the derivation it is clear that the expression of Supplementary Eq. (9) is valid for any value of J_{g} when $\langle \hat{c}^\dagger_{\mathrm{g},i}\hat{c}_{\mathrm{g},j}\rangle \ll \langle \hat{n}_{\mathrm{g},i}\rangle$, if we do not take a superfluid component into consideration. This means that, even for localized states, quantum fluctuations resulting from the second-order round-trip tunneling cause the broadening of each spectral peak. Note that this broadening effect is independent of i, m, because the relevant term of spectral variance $\sigma^2_{\mathrm{NDS},\alpha} = \sum_i \sum_{\mathbf{d}} |\delta J_{\alpha,\mathbf{d}}|^2 \langle \hat{n}_{\mathrm{g},i}\rangle / \sum_i \langle \hat{n}_{\mathrm{g},i}\rangle$ reduces to a constant $\sum_{\mathbf{d}} |\delta J_{\alpha,\mathbf{d}}|^2$.

Spectra for the superfluid

Next, let us consider the opposite limit of $n_{\mathrm{NF}} \sim 0$, where we neglect $I_{\mathrm{NF},\alpha}(\nu)$. To obtain the spectrum $I_{\mathrm{SF},\alpha}(\nu)$, we need to perform the summation of all of the excited states in Eq. (5) in the main text. However, except for $U_{\mathrm{gg}} = 0$, it is very difficult to calculate the summation. We can expect the moment expansion mentioned above to allow us to obtain a reasonably valid expression of the spectra instead of the rigorous sum of all excited states.

Here, we assume that $I_{\mathrm{SF},\alpha}(\nu)$ can be well described by the following form:

$$I_{\mathrm{SF},\alpha}(\nu) = n_{\mathrm{SF}} \frac{\exp\Big(- h^2(\nu - p_{\mathrm{SF},\alpha})^2/(2\sigma^2_{\mathrm{SF},\alpha})\Big)}{(\sigma_{\mathrm{SF},\alpha}\sqrt{2\pi})}, \tag{11}$$

where the parameters $p_{\mathrm{SF},\alpha}$ and $\sigma_{\mathrm{SF},\alpha}$ are not free fitting parameters and are properly determined from the sum rules of Supplementary Eq. (3) with ℓ =1 and 2. Namely, $M_{1,\alpha} = n_{\mathrm{SF}} h p_{\mathrm{SF},\alpha}$ and $M_{2,\alpha} = n_{\mathrm{SF}} \times (h^2 p^2_{\mathrm{SF},\alpha} + \sigma^2_{\mathrm{SF},\alpha})$. It is also simple to confirm that the expression Supplementary Eq. (11), and especially the weights, satisfies the zero-th order sum rule. We assume that the $I_{\mathrm{SF},\alpha}(\nu)$ has a single peak for a certain α orbital-exchange excitation, although in principle a multi-peak structure is possible owing to the different excitation energies for different i values. We should note that such a structure is properly included by considering the second order moment given in Eq. (11) in the main text and the resulting spectral width of $\sigma_{\mathrm{SF},\alpha}$, which is related to the number fluctuation caused by the superfluid component: $\sigma_{\mathrm{SF},\alpha} = \delta U_\alpha \sqrt{n_{\mathrm{SF}}}$. Note that we define number fluctuations as $\Delta n^2 = \langle \hat{n}^2_{\mathrm{g},i}\rangle - \langle \hat{n}_{\mathrm{g},i}\rangle^2$, and we obtain $\Delta n_{\mathrm{SF}} = \sqrt{n}_{\mathrm{SF}}$ by using a mean-field approximation. The above expression corresponds to Eq. (9) in the main text.

Spectra for coexisting region

Finally, we explain the formulation for general cases of $n_{\mathrm{SF}} \neq 0$ and $n_{\mathrm{NF}} \neq 0$, where the NF and SF atoms coexist. On the basis of the binary fluid approximation, we calculate the spectra for the NF and the SF states separately.

First, we calculate $I_{\mathrm{NF},\alpha}(\nu)$ in the same way as we calculate the spectra for normal fluids, which is given in Supplementary Eq. (9), but $I_{\mathrm{NF},\alpha}(\nu)$ is now affected by the mean-field potential originating from the interaction with the SF atoms. Thus, by introducing the effective mean-field potential term V_{SF} we here consider the following effective Hamiltonian for the NF atoms in the ground state:

$$\hat{\mathcal{H}}_{\mathrm{NF}} = \sum_i (V_{\mathrm{g},i} + V_{\mathrm{SF},i})\hat{n}_{\mathrm{NF},i} + U_{\mathrm{gg}} \sum_i \frac{\hat{n}_{\mathrm{NF},i}(\hat{n}_{\mathrm{NF},i}-1)}{2}. \tag{12}$$

Here in Supplementary Eq. (12), there is no explicit term for hopping proportional to J_{g} but it is effectively incorporated in the mean-field interaction $V_{\mathrm{SF},i}$, which is valid under the Gutzwiller approximation. The second order hopping is also properly included in the width of the spectra $\sigma_{\mathrm{NDS},\alpha}$, as already discussed in Methods and Supplementary Information. The potential term $V_{\mathrm{SF},i}\hat{n}_{\mathrm{NF},i}$ is written as $U_{\mathrm{gg}} n_{\mathrm{SF},i}\hat{n}_{\mathrm{NF},i} + \delta\mu_i \hat{n}_{\mathrm{NF},i}$, where the first term represents the interaction between the SF and NF atoms within a mean-field level. We determine $\delta\mu_i$ so that the number of NF atoms evaluated with the effective Hamiltonian Supplementary Eq. (12) will give the correct values of $\langle \hat{n}_{\mathrm{g},i}\rangle - |\langle \hat{c}_{\mathrm{g},i}\rangle|^2$ evaluated with the original Hamiltonian Eq. (1) in the main text. Note that $\delta\mu_i \sim 0$ for $n_{\mathrm{SF},i} \gg n_{\mathrm{NF},i}$ or $n_{\mathrm{SF},i} \ll n_{\mathrm{NF},i}$. After determining the $V_{\mathrm{SF},i}$ in this way, we can obtain $I_{\mathrm{NF},\alpha}(\nu)$ with Supplementary Eq. (9) by diagonalizing the effective Hamiltonian Supplementary Eq. (12) with the finite temperature Gutzwiller approximation.

After obtaining $I_{\mathrm{NF},\alpha}(\nu)$, we can determine $I_{\mathrm{SF},\alpha}(\nu)$ with the same spectral shape as Supplementary Eq. (11) but with different $p_{\mathrm{SF},\alpha}$ and $\sigma_{\mathrm{SF},\alpha}$ values, which are determined by using the sum rule relation Supplementary Eq. (3). These can be rewritten as:

$$h p_{\mathrm{SF},\alpha} n_{\mathrm{SF}} = M_{1,\alpha} - \int h\nu I_{\mathrm{NF},\alpha}(\nu) d\nu, \tag{13}$$

$$\sigma^2_{\mathrm{SF},\alpha} n_{\mathrm{SF}} = M_{2,\alpha} - h^2 p^2_{\mathrm{SF},\alpha} n_{\mathrm{SF}} - \int (h\nu)^2 I_{\mathrm{NF},\alpha}(\nu) d\nu. \tag{14}$$

Since $M_{\ell,\alpha}$ can be calculated from thermodynamic quantities alone, as shown in Eqs. (10) and (11) in the main text, respectively, we can readily obtain $p_{\mathrm{SF},\alpha}$ and $\sigma_{\mathrm{SF},\alpha}$ from Supplementary Eqs. (13) and (14).

Supplementary Note 10: Orbital changing excitations and kinetic energy shifts and broadening

We discuss the effects of the kinetic energy contributions appearing in the spectrum, which are one of the characteristics of laser spectroscopy not observed in RF spectroscopy [8]. A prominent example is the orbital changing excitation, which can be found in spectra at $\nu > 0$. From the definition of the excitation probability $|\eta_\alpha|^2$ with $\eta_\alpha = \int d\mathbf{r} \Psi_{\mathrm{e},i\alpha}(\mathbf{r}) e^{i\mathbf{k}_{\mathrm{ex}}\cdot(\mathbf{r}-\mathbf{r}_i)} \Psi_{\mathrm{g},i}(\mathbf{r})$, we find that this excitation is caused by the momentum transfer $\mathbf{k}_{\mathrm{ex}}$ from the excitation laser. The first moment in Eq. (10) in the main text shows that the kinetic (band gap) energy Δ_α should be given by the lasers when orbital changing excitations occur.

The first moment $M_{1,\alpha}$ in Eq. (10) further shows that the spectral peak positions are affected by the kinetic energy. For convenience, we rewrite one of the terms in Eq. (10), $\sum_{i,\alpha,\mathbf{d}} \delta J_{\alpha,\mathbf{d}} \langle \hat{c}^\dagger_{\mathrm{g},i} \hat{c}_{\mathrm{g},i+d}\rangle$, based on the Bloch band pictures by using Fourier transformation: This term is rewritten as $\sum_{\mathbf{k}} \delta\varepsilon_{\alpha,\mathbf{k},\mathbf{k}_{\mathrm{ex}}} n_{g,\mathbf{k}}$, where $n_{g,\mathbf{k}}$ is the momentum distribution of the electronic ground state atoms, and $\delta\varepsilon_{\alpha,\mathbf{k}}$ defined by $\varepsilon_{e\alpha,\mathbf{k}+\mathbf{k}_{\mathrm{ex}}} - \varepsilon_{g,\mathbf{k}}$ is the kinetic energy difference including momentum transfer effects. Here $\varepsilon_{g,\mathbf{k}}$ and $\varepsilon_{e\alpha,\mathbf{k}}$ are the dispersions of the electronic ground state atoms in the lowest orbital and of the electronic excited atoms in the α-orbital, respectively. Note that $\delta\varepsilon_{\alpha,\mathbf{k}}$ does not include the band gap Δ_α, whereas $\delta V_{i,\alpha}$ in Eq. (10) includes this term. By considering the spectral mean value given by $M_{1,\alpha}/M_{0,\alpha}$, we obtain the kinetic energy shifts of the spectral peak positions as $\sum_{\mathbf{k}} \delta\varepsilon_{\alpha,\mathbf{k}} n_{g,\mathbf{k}}/n_{\mathrm{tot}}$. We should note that, quantitatively, the SF states in shallow lattices are significantly affected by this kinetic energy shift, while the MI states in deep lattices are hardly affected. For simplicity, by assuming that the SF atoms are condensed into the state at the bottom of the dispersion $n_{g,\mathbf{k}} = \delta_{\mathbf{k},\mathbf{0}} n_{\mathrm{SF}}$, we can obtain a kinetic energy shift of $\delta\varepsilon_{\alpha,\mathbf{0}} n_{\mathrm{SF}}/n_{\mathrm{tot}}$, where $\delta\varepsilon_{\alpha,\mathbf{0}} = -2\sum_{\mathbf{d}} (J_{e,\alpha} e^{i\mathbf{d}\cdot\mathbf{k}_{\mathrm{ex}}} - J_g)$. Using this expression, we can roughly estimate the kinetic energy shift for $5E_r$ as about $+1.7$ kHz for $\alpha = 0$ and about -4.8 kHz for $\alpha = 1$, where we use $\mathbf{k}_{\mathrm{ex}} \sim (0.74\pi, 0.74\pi, 0)$. The different signs of the kinetic energy shifts for $\alpha = 0$ and 1 result from the fact that the sign of $J_{e,1}$ is opposite to that of $J_{e,0}$. Furthermore, as mentioned in the main text, the spectral broadening of $I_{\mathrm{NF}}(\nu)$ is caused by the kinetic energy contributions. We find that such effects are important to quantitative agreements between experimental data and numerical simulations in particular for shallow lattices with $V_L \lesssim 8E_r$.

Finally, we comment on the difference between laser and RF spectroscopy. By comparing the wavelenghts of microwaves with the lattice constant a_L, we find that the transfered momentum $\mathbf{k}_{\mathrm{ex}}$ is negligibly small in microwave

spectroscopy by comparison with lattice wavevector $2\pi/a_L$, where a_L is half of the wavelength of the lattice lasers. Thus, the kinetic contributions are negligible in RF spectroscopy. In fact, from the orthogonality of the Wannier functions, we can easily determine that the excitation probabilities are given by $|\eta_{\alpha=0}|^2 = 1$ and $|\eta_{\alpha\neq 0}|^2 = 0$ for $\mathbf{k}_{\rm ex} = 0$. We can thus conclude that microwave spectroscopy is in a perfect Lamb-Dick regime [9]. The kinetic energy shifts and broadenings are also negligible in such spectroscopy with a long wavelength.

Supplementary Note 11: Spectral decomposition of superfluid and normal fluid contributions at various lattice depths

We show numerical results for the superfluid and normal fluid contributions in the excitation spectra in Supplementary Fig. 6 for a lattice depth $V_{\rm L}$=1-15$\rm E_r$ with an initial temperature $T_{\rm ini}$ of 95 nK. In addition, the calculated SF and NF atom density distributions in the trap is plotted along with the entropy distribution in Supplementary Fig. 7 for a lattice depth $V_{\rm L}$=1-15$\rm E_r$. Supplementary Fig. 7 shows the dip structure of the entropy distributions in deep lattices ($> 12\rm E_r$) and the shoulder-like structure of the number distributions in the corresponding lattice sites with the entropy dip structures, which are the signatures of the crossover from the NF to MI. For the NF states at higher temperatures, there are no dip structures in the entropy distributions, and number distributions show smooth curves. We can clearly see a great change in the contributions from each component, which coincides with the change in the numerically calculated atomic distributions, as the lattice depth is changed. In addition, we show the numerical results obtained with different initial temperatures in Supplementary Fig. 8. The results with the highest initial temperature $T_{\rm ini}$(=150 nK) show that the ratio between the peak intensities for the multiply and singly occupied sites is very different from the results with the lower initial temperatures. This is because large thermal fluctuations induce wide and dilute density distributions of atoms. As a result, the peak corresponding to single occupancy has a relatively large intensity.

Supplementary Note 12: Checking sum rule relations

Here we confirm the validity of using the sum rule relations in the present study, which is an important basis for our theoretical analysis. We evaluate the spectral moments $M_\ell = \sum_\alpha |\eta_\alpha|^2 M_{\ell,\alpha} = \int (h\nu)^\ell I(\nu) d\nu$. The right-hand side is evaluated by the theoretically calculated correlation functions for the ground state in thermal equilibrium before excitation. In Supplementary Fig. 9, we plot M_1 and M_2 from the experimentally obtained spectra (filled circles) as a function of the lattice depths. At all the lattice depths that cover the weakly to strongly interacting regimes, the evaluated M_1 values agree reasonably well with the numerical results (diamonds), as shown in Supplementary Fig. 9**a**. The M_2 values also show reasonable agreement below 8$\rm E_r$, as is shown in Supplementary Fig. 9**b**. The discrepancy with M_2 in Supplementary Fig. 9**b** at a relatively deep lattice depth (9-15$\rm E_r$) can be attributed to the contributions from the triple and higher occupancies in the spectra. We should note that we attribute the applicability of the sum rule relations in our high-resolution spectroscopy to our novel experimental conditions, namely the sufficiently weak excitation and the limited range of the energy spectra in ultracold atomic systems isolated from the environment, which is difficult to realize in condensed matter systems because of the complicated multi-band structure.

SUPPLEMENTARY REFERENCES

[1] Kato, S., Shibata, K., Yamamoto, R., Yoshikawa, Y. and Takahashi, Y. Optical magnetic resonance imaging with an ultra-narrow optical transition. Applied Physics B **108**, 31–38 (2012).

[2] Killian, T. 1S-2S spectrum of a hydrogen Bose-Einstein condensate. Physical Review A **61**, 033611 (2000).

[3] Yamaguchi, A., Uetake, S., Kato, S., Ito, H. and Takahashi, Y. High-resolution laser spectroscopy of a Bose–Einstein condensate using the ultranarrow magnetic quadrupole transition. New Journal of Physics **12**, 103001 (2010).

[4] Uetake, S., Murakami, R., Doyle, J. M. and Takahashi, Y. Spin-dependent collision of ultracold metastable atoms. Phys. Rev. A **86**, 032712 (2012).

[5] Yamashita, M. and Jack, M. W. Mott-insulator shells in the three-dimensional Bose-Hubbard model with harmonic confinement. Physical Review A **79**, 023609 (2009).

[6] Inaba, K. and Yamashita, M. Theoretical Analysis on Spectroscopy of Atomic Bose-Hubbard Systems. Preprint at http://arxiv.org/abs/1507.06399.

[7] Hazzard, K. R. A. and Mueller, E. J. Hyperfine spectra of trapped bosons in optical lattices. Physical Review A **76**, 063612 (2007).
[8] Campbell, G. K. et al. Imaging the Mott Insulator Shells by Using Atomic Clock Shifts. Science (New York, N.Y.) **313**, 649–652 (2006).
[9] Dicke, R. H. The effect of collisions upon the doppler width of spectral lines. Phys. Rev. **89**, 472–473 (1953).
